# Bidirectional rotational antagonistic shape memory alloy actuators for high-frequency artificial muscles

**DOI:** 10.1038/s41598-025-93209-9

**Published:** 2025-03-17

**Authors:** Rawan Barakat, Susanne-Marie Kirsch, Felix Welsch, Paul Motzki

**Affiliations:** 1 Smart Material Systems, Department of Systems Engineering, Saarland University, 66123 Saarbruecken, Germany; 2 ZeMA-Center for Mechatronics and Automation Technology, 66121 Saarbruecken, Germany

**Keywords:** Shape memory alloy, Actuator, High-frequency, Bio-inspired, Robotic, Artificial muscle, Biomimetics, Electrical and electronic engineering, Engineering, Biomedical engineering

## Abstract

Shape memory alloys (SMA) are commonly utilized in compact actuators due to their high energy density, meaning possible work output in relation to their weight and volume. Their application area is limited by their poor dynamic behavior, caused by the thermal activation characteristics of SMA materials. Typical actuation frequencies of SMA-based actuators range from 1 Hz to 10 Hz. In this work, we introduce an actuator system architecture, termed bidirectional rotational antagonistic (BIRAN) SMA actuator, which uses bundles of thin SMA wires to generate repeated rotational movement at frequencies up to 200 Hz. This marks a new frequency record for electrically activated SMA wire-based actuator systems. The high frequency reported results from the combination of mechanical design, electronics, and control strategy. We describe the fabrication techniques and the power electronics development and demonstrate the system performance through a systematic experimental study. A bio-inspired robotic wing-flapping joint illustrates the expansion of possible SMA-based application areas, pushing the dynamic limitations of this actuator technology.

## Introduction

In recent decades, increased interest in recreating motion types from nature has been observed^1–3^. The research field is known as bionics, biomimetic, or bio-inspired robotics. Using artificial muscle materials allows creating soft robotics with high adaptability to natural motion types. In underwater applications, ionic polymer-metal composites (IPMC) are commonly used, as it is provided by their working principle. Dielectric elastomer actuators (DEA) are known for their high material strain, high frequencies up to loudspeaker applications and their inherently soft material characteristics^4–7^. Shape memory alloys (SMA) are primary candidates for artificial muscles in powerful and compact robots due to their high energy density and muscle-like contractile behavior in wires, especially in the antagonistic configuration^8–10^. Due to the wide variety of possible alloys, available geometries (wires, films, springs, or ribbons), and manufacturing methods, SMA cover a wide range of applications^8,9,11^. While their high energy density makes them appealing for powerful, compact and lightweight applications, such as flying robots, the relatively low operation frequency related to the thermal activation characteristics counts as a limiting factor^4^.

The thermally activated SMA are classified based on the type of heating into external heating (convection) and internal heating (resistive heating via applying electrical power). After heating the SMA to a specific temperature, it starts to transform to the high temperature phase (austenite) and it reverts to the low temperature phase (martensite) upon cooling. The transformation between austenite and martensite leads to a deformation of the SMA element on a macroscopic level. By repeated cycles of heating and cooling and thereby repeated transformation between austenite and martensite, an actuator can be created. To achieve an accurately defined actuation upon cooling, the SMA material must be pre-strained, for example, using a mass, spring, or another SMA component^10^. Besides the accuracy, the dynamic behavior of the SMA actuator is highly relevant for many applications, especially in the bio-inspired robotics, medical field such as robotic surgery or active implants for promoting the healing of bone fracture^9,12,13^.

The work’s main contribution is the expansion of the potential and the application area of SMA actuators by improving their dynamic behavior. This improvement in dynamic behavior is achieved through a combination of a specific actuator design (BIRAN), a tailored power electronics architecture, and effective control implementation.

By observing reported SMA actuators with operation frequencies higher than 10 Hz, it is observable, that SMA thin-films are more capable of high-frequency applications^14–17^, compared to SMA thick-films and SMA wires. SMA thin-film actuators are mainly present in micro- and nanoactuators^15–19^. SMA wires and SMA thick films seem to be present in similar frequency ranges below 50 Hz^20–27^. SMA wire actuators are highly present in bio-inspired robots^24–26^, due to the muscle-like contractile behavior. All surveyed actuators that operate in the kHz range share a common characteristic of possessing exceedingly small volumes. Knick et al.^17^ reported 3 kHz operation frequency of a Micro-Electro-Mechanical System (MEMS) bimorph actuator based on ultra-thin SMA films within the nanoscale range. A similar work reported 1 kHz using 600 nm SMA thin-film^16^. The operating frequency of 1 kHz was also reported using an SMA-based nanotweezer^28^. The highest frequency reported using SMA wires originated from a MEMS actuator with 25 $\upmu$m wires manufactured with diamond structure to reduce the volume and thereby enhance the surface-to-volume ratio.

**Figure 1:**
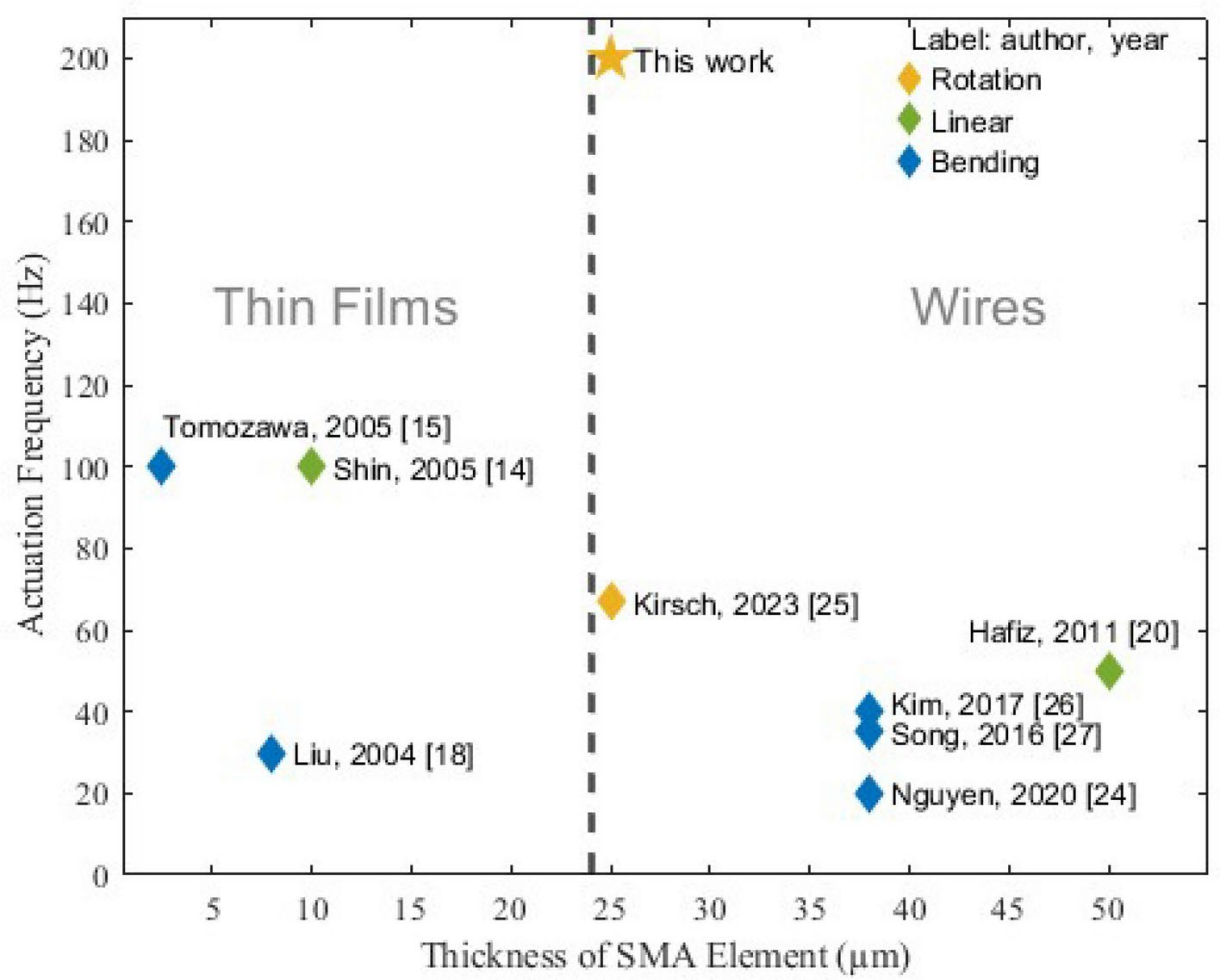
Overview of non-MEMS SMA-based actuators at operational frequencies higher than 10 Hz. The overview shows the type of motion and the geometry of the used SMA element for each actuator.

Figure 1 gives an overview of non-MEMS SMA actuators with operation frequencies higher than 10 Hz. The overview considers SMA thin films and SMA thin wires due to their capability to operate at high frequencies. It is known that the surface-to-volume ratio of the SMA element is one of the main factors affecting its dynamic behavior.

In the surveyed works in Fig. 1, different approaches have been followed to realize high frequencies. For example, in work^26^, a high-frequency flapping motion has been realized by antagonistic bending of 38 $\upmu$m thick SMA wires. With around 40 Hz, the former highest record of 35 Hz from their former work^27^ was surpassed. The high surface-to-volume ratio, along with the antagonistic arrangement of the wires, contributes to the high frequency actuation. Using the same wire thickness of 38 $\upmu$m and a leaf spring, a crawling SMA microactuator was realized^24^. At an actuation frequency of 20 Hz, the microactuator generates fast jumps. Similar to the previously mentioned works, the achieved results were mainly based on considerations of mechanical design. No active cooling methods were employed. In^25^, an SMA actuator consisting of two bundles of 25 $\upmu$m SMA wires in an antagonistic configuration was developed to replicate the wing-flapping motion of bats. A novel manufacturing method was developed to overcome the challenging handling of thin SMA wires. The reported operation frequency was around 67 Hz. All mentioned applications belong to the biomimics field. In the last SMA wire-based actuator, in^20^, a 50 $\upmu$m wire was used to generate a micro-vibration actuator for touch screens with tactile feedback. For this purpose, there is no demand for high displacement.

In two of the mentioned applications with thin SMA films, a rare SMA material, NiTiPd, with a high phase transformation temperature was utilized to decrease the cooling time^15,18^. In^14^, an 8 $\upmu$m SMA thin film was used to realize a compact actuator pump, in which the generated liquid flow was utilized to actively cool the NiTi SMA membrane.

The developed BIRAN SMA actuator in this work represents the SMA actuator with the highest frequency reported in the non-MEMS fields, it surpasses the operation frequencies reported for SMA thin film actuators. Using unmodified commercial Nickel-Titanium (NiTi) wire with a diameter of 25 $\upmu$m, the BIRAN actuator is fabricated using a specific bundling method. Together with the developed power electronics and control strategy, the BIRAN actuator reaches a repeated actuation frequency of 200 Hz.

**Figure 2:**
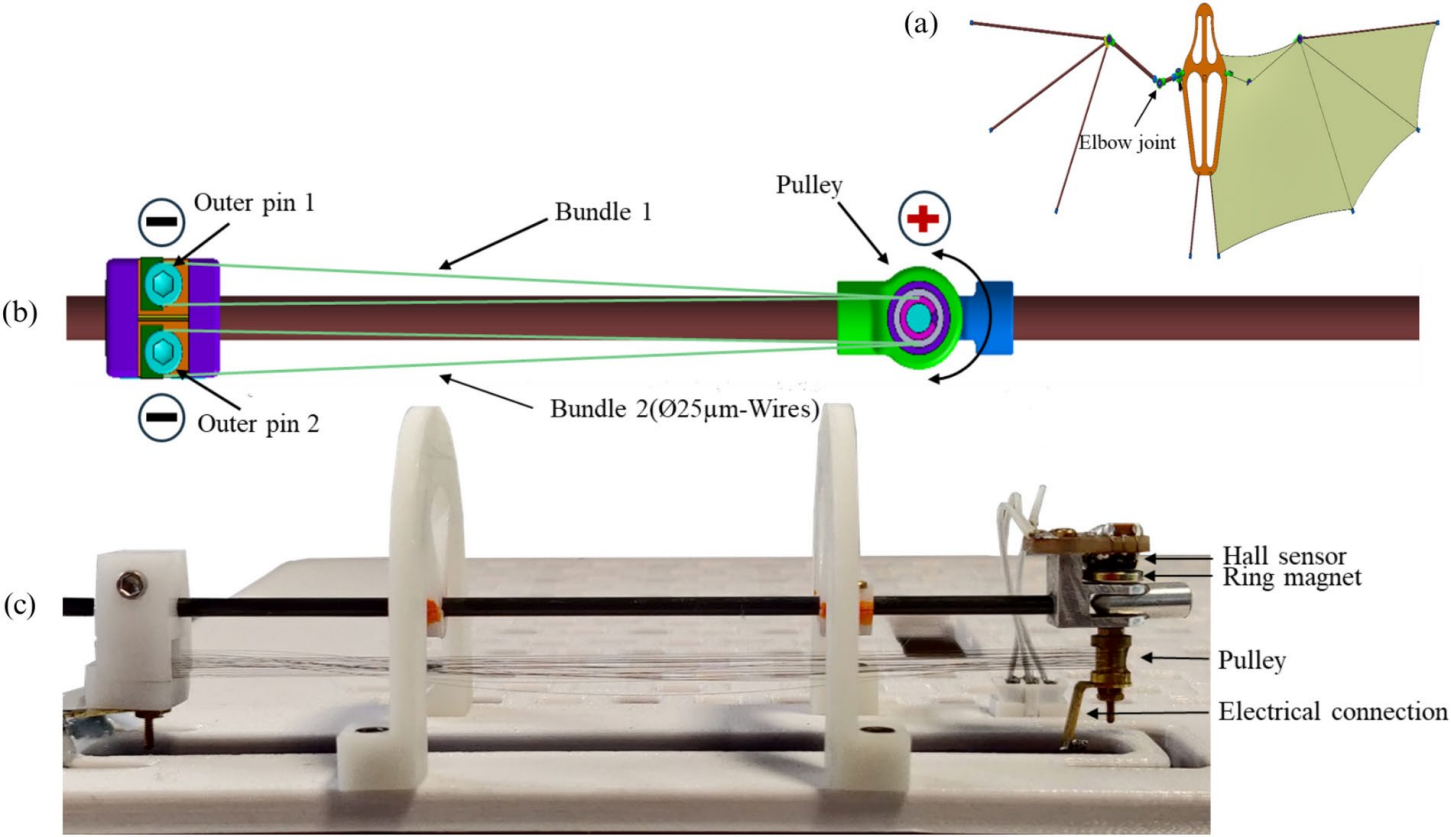
The assembled BIRAN actuator (**a**) built in the wing of the bat robot, (**b**) CAD-Model of the SMA actuator, (**c**) a photo of the SMA actuator in the experimental characterization setup.

The presented work is the continuation of a previous project that aimed to replicate the flying motion of bats^25,29–31^, shown in Fig. 2.a. The actuator design supports the cooling process passively as it consists of thin SMA wires with a diameter of 25 $\upmu$m allowing a high surface-to-volume ratio and thus fast convective cooling. To maintain sufficiently high force, a bundle of 20 wires is used. The novel manufacturing method uses one continuous SMA wire to create two bundle segments in an efficient and reproducible assembly step. Furthermore, forced air is applied to replicate the aerodynamic conditions, which also serves as an active cooling method and shortens the cooling time. To support the heating process, higher current pulses are applied to reach the transformation temperature within a shorter period of time. At the same time, decreasing the duration of the heating process saves time for the cooling process. First measurement results are presented in^25,29^, a maximum frequency of around 67 Hz has been measured with a rotation angle of $43.9~^\circ$ by applying 4.3 A for 2.5 ms.

However, in nature, there is a wide range of flapping frequencies present, butterflies^32^ and bats^33^ with around 10 Hz in the low frequency range, higher flapping frequencies are present in some insects like honeybee^34^ with 230 Hz and Culicidae with a frequency of up to 480 Hz^35^.

The capability of the BIRAN actuator to replicate higher flapping frequencies by applying higher currents is proven in this work. For this, an electronic circuit is developed to fully utilize the potential of the actuator regarding its actuation frequency. The BIRAN actuator aims to expand the application frequency range of SMA actuators, especially flying SMA robots. It shows the capability of electrically activated SMA actuators for high operation frequencies, when taking into account the mechanical, electrical, and control aspects.

## Working principle

The BIRAN actuator consists of two SMA bundle segments that can be alternately activated in an antagonistic manner to generate a rotational motion. The bundles are mechanically and electrically connected to a common pulley and two outer pins, as shown in Fig. 2b. Both outer pins and the middle pulley are machined of electrically conducting material (brass). At the pulley, both bundles share a common electric potential. As long as one of the outer pins is connected to a higher/lower electrical potential, an electrical current flows through the according SMA bundle owing to the resulting potential difference. The flowing current heats the SMA bundles and after it reaches a specific temperature, it starts to undergo a phase transformation from martensite to austenite. The bundle contracts and applies torque at the pulley causing its rotation in the direction of the heated bundle segment. By alternately activating the SMA bundle segments they operate in an antagonistic fashion, causing bidirectional rotational movement, like a biological joint. This type of arrangement is known as antagonistic and it contributes, besides the geometrical considerations, to enhancing the dynamic behavior of the SMA actuator without a degradation in the net force^36^. Figures 2b and 2c show a developed and fabricated BIRAN actuator prototype. Video material of the active prototype is provided in the supplementary content.

## Results and discussion

### Angle measurements

**Figure 3:**
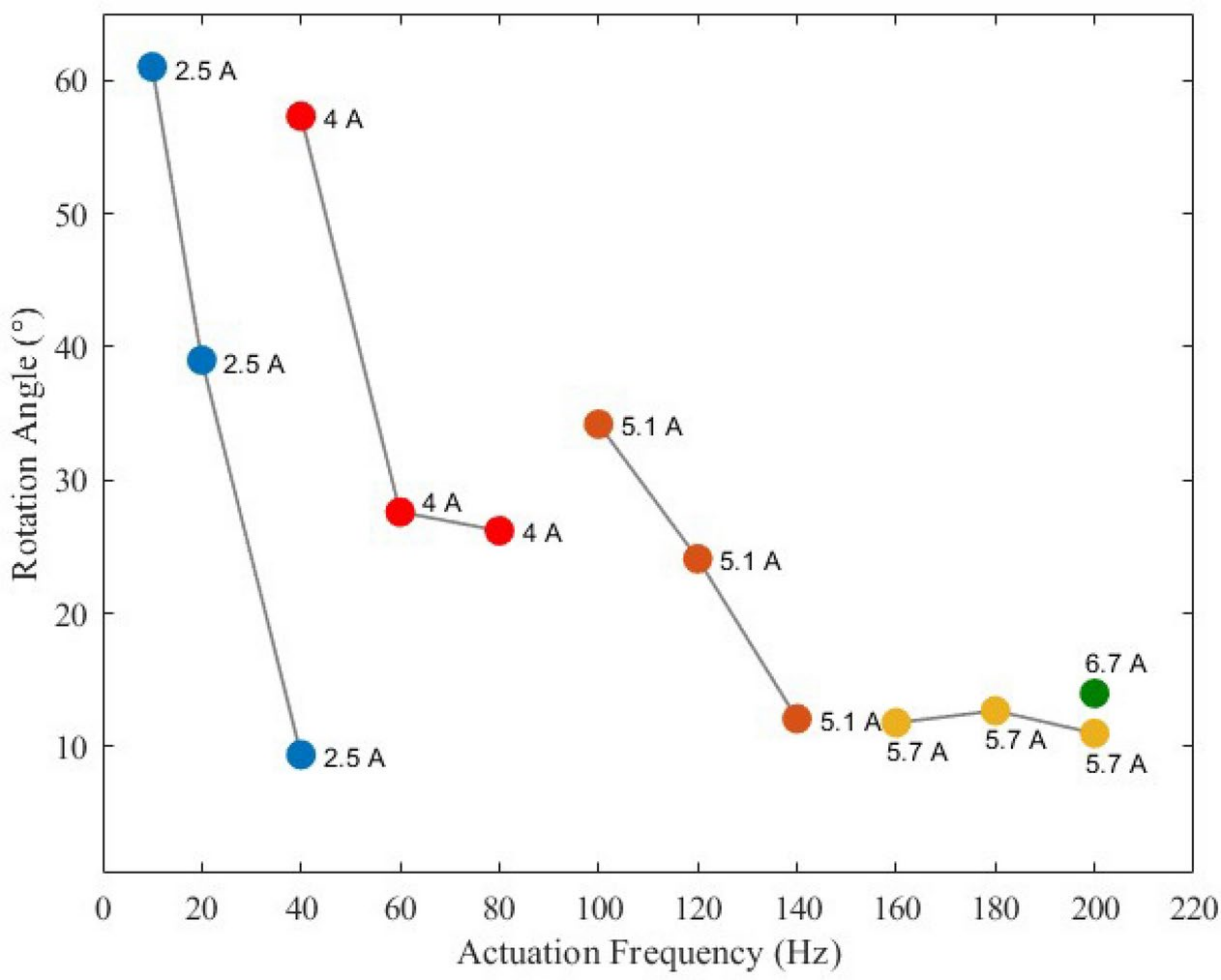
Measured angles at different frequencies and different current values.

Figure 3 shows an overview of the conducted measurements at different frequencies between 10  and 200 Hz. The different colors indicate different current values, blue, red, orange, and yellow for 2.5 A, 4 A, 5.1 A, and 5.7 A respectively.

One activation cycle of an SMA element includes the heating and the cooling time. The most time-consuming part is the cooling. The cooling behavior is the main limiting factor in SMA actuators. Under the same cooling conditions, increasing the frequency reduces the time to cool down. However, increasing the current shortens the heating time and saves more time for cooling. Up to a specific limit, increasing the frequency doesn’t lead directly to a lower rotation angle, as it is shown by the measurements at 60 Hz and 100 Hz. The measurement results are provided in the supplementary content. It depends on the distribution of the heating and cooling time. If the frequency reaches a limit, at which the period is shorter than twice the required cooling time, due to the antagonistic configuration, a decrease in the stroke can’t be avoided, as long as the cooling conditions are not improved. Additionally, if the applied energy is not enough to finish the phase transformation from martensite to austenite, a decrease in the reached displacement will be measured. At 2.5 *A* the measured rotation angle decreases from $61^{\circ }$ to $9.4^{\circ }$ as the frequency rises from 10 to 40 Hz. After increasing the current value at 40 Hz from 2.5 to 4 A, the measured rotation angle reaches $57.3^{\circ }$. The highest applied current value is 6.7 A. At 200 Hz a rotation angle of around $14^{\circ }$ is measured. Higher rotation angles are expected by applying higher currents.

Table 1 shows further details for several experiments, including the heating time, the cooling time, and the applied airflow of the fan. In previous work on the BIRAN actuator^25^, it has been shown that an assumption of the adiabatic heating process, meaning no energy is lost to the environment during the SMA activation, is valid for an activation time below 5 *ms*. The column $I_{\text {calculated}}$ is calculated based on previous experiments and according to Eq. (1) under the assumption of an adiabatic heating process. By applying the $I_{\text {calculated}}$ values, a full phase transformation from martensite to austenite within the given activation time $t_{\text {on}}$ is expected. In these experiments, lower current values are applied due to the power limitation of available power supplies. An in-depth discussion follows in “Limitations and future work”.

For a solid statement on the lifetime and fatigue behavior, comprehensive experiments should be conducted. However, at this stage, more than 30 experiments have been performed on the BIRAN actuator, each with 5 rotation cycles, without noticeable degradation in the performance.

**Table 1:** An overview of conducted experiments at different frequencies between 10 and 200 Hz and reached angle.

Frequency (Hz)	t_on (ms)	t_off (ms)	I_applied (A)	I_calculated (A)	Angle ($^\circ$)	Airflow (m/s)
10	10	40	2.5	2.5	61	8
40	2.5	10	4	4.4	57.3	10.5
100	1	4	5.1	7.9	34.2	10.5
200	0.5	2	6.7	9.8	14	10.5

### Resistance measurement

**Figure 4:**
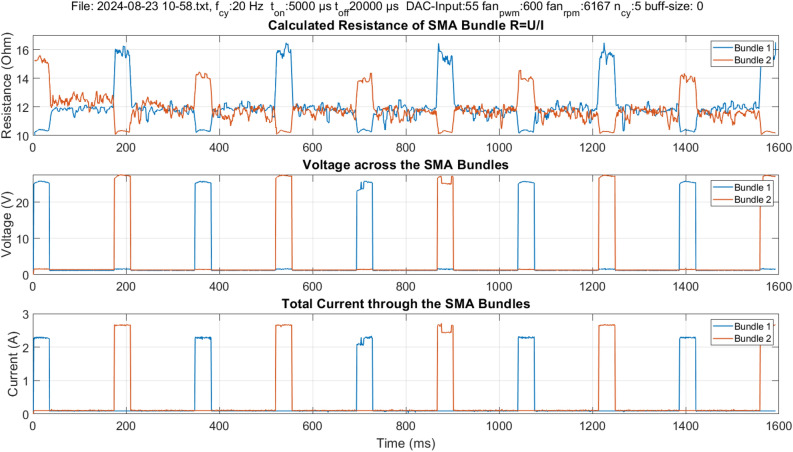
Example of resistance measurement according to the four wires measurement method. The resistance values of the SMA bundles are calculated from the measured voltages and currents. The shown experiment is performed at 20 Hz with 5 ms on-time and 20 ms off-time per bundle.

The resistance measurement is performed using the developed electronic aiming to utilize the self-sensing property of SMA. The self-sensing describes the ability of the material to serve as a sensor and actuator at the same time, in SMA the sensing property appears in the form of resistance change during phase transformation^10^. Figure 4 shows in the upper part the change in the resistance value throughout the actuation cycles. One bundle is indicated with blue, the other with orange. Following the resistance development of the blue-indicated bundle for example: when the control current flows through the bundles, a higher voltage drop is measured across it, as shown in the middle and lower part of Fig. 4. As the SMA bundle contracts by thermal activation, its resistance decreases and it reaches a minimum value during a cycle, as shown in the upper part of Fig. 4. After the current pulse is over, the SMA bundle starts to cool down and its resistance changes going back to the middle line at around $12~\Omega$. In the second half cycle, when the antagonistic bundle is activated, it contracts causing the blue-indicated bundle to elongate. An increase in resistance value is observed. The resistance value is calculated from the measured currents and voltages. The measurement shown is conducted at 20 Hz with a set current value of 2.5 A.

### Power electronics

The accuracy of the applied current is crucial to avoid overheating the SMA bundle and therefore to avoid an accelerated fatigue process or, in the worst case, instant damage. The accuracy is defined here as the difference between the expected and the measured value.

The current source shows an accuracy of 99% evaluated with a highly accurate multimeter, with 1% error. The measured error of the current source lies within the error range of the measuring tools. On the side of the current source itself, the accuracy is mainly limited by the power shunt resistor which has a 1% tolerance. Regarding the rising time of the current signal, an upper limit is defined at $10\%$ of the activation time $t_{on}$. The experiments show a dependency of the rising time on the set current value and the load, i.e. its resistive and inductive loads. For a resistive load, a rising time below $1~\mu s$ is measured.

## Methods

### Actuator design

**Figure 5:**
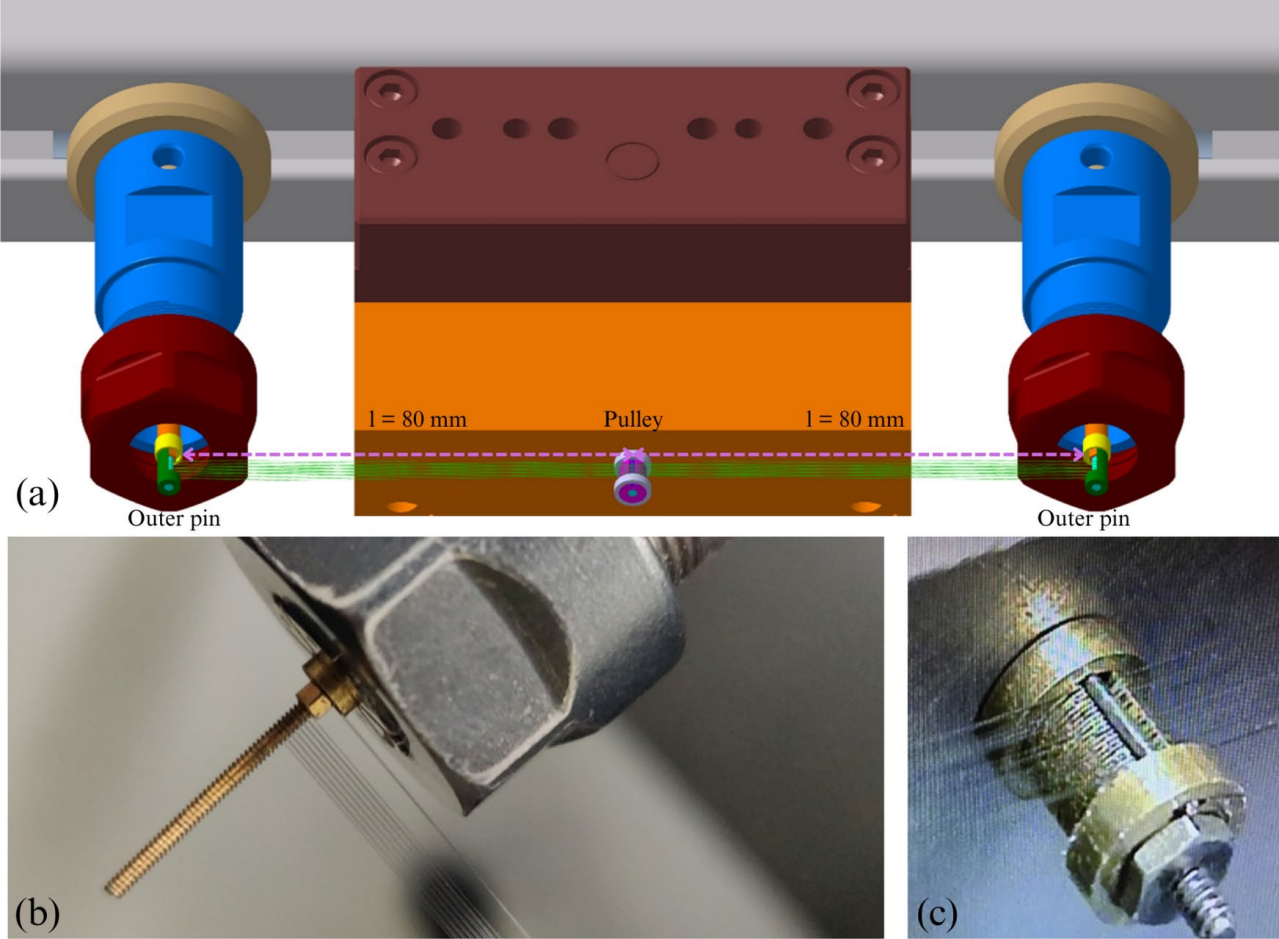
Manufacturing process of the SMA actuator using a custom-built winding machine.

The BIRAN actuator is constructed out of a single continuous 25 $\upmu$m thick SMA wire used to wind a bundle of 20 wires. The resulting micro-wire bundle is then split into two symmetrical segments. To ensure repetitive and reliable manufacturing of the micro-wire bundle actuator, a novel winding machine was developed and patented^31^. As shown in Fig. 5, all three pins of the actuator, the middle pin with the pulley, and the two outer pins are fixed into the winding machine. Their position is adjustable, here it is set to 80 mm between each outer pin and the middle pin with the pulley, which defines the length of the two bundle segments. The outer pins have a diameter of 1 mm. The vertical distance between the wires of the bundle is determined by the thread of the outer pins, here 0.2 mm. The maximum distance between both bundles is 0.7 mm, which is given by a 3D-printed clamping mechanism (purple part in Fig. 2.b). The pulley in the middle has a height of 2.625 mm and a radius of 1.5 mm. With its thread, it allows a wire spacing of 0.125 mm.

In an automated winding process, the mentioned parameters are added to the control software, which controls all three motors of the winding machine. The user is responsible for connecting one end of the continuous wire to one outer pin using a nut. Then the motors start to work in a synchronous way. One motor rotates the set shown in the upper part of Fig. 5 with the three pins and forms thereby the bundle around the outer pins. As the bundle is constructed of one continuous wire, another motor rotates the wire coil synchronously with the first motor and maintains thereby a constant wire stress during the winding process. This ensures a reliable thermo-mechanical behavior of the actuator as introduced in^25^. The third motor is a linear step motor with a motion axis vertical to the bundle winding plane, it directs the wire coming from the wire coil to be located correctly along the outer pin threads in each cycle in the winding process. After 10 winding cycles, it results in a bundle of 20 wires. Before cutting the continuous wire, the bundle is secured at both outer pins and the pulley using a conductive resin. In the next step, a protective brass cover is added to both outer pins. In the last step, the bundle is released from the winding machine and bent around the pulley to create two bundle segments.

For the angle measurement, a diametral ring magnet is attached to the middle pin above the pulley, allowing it to rotate along with the pulley. A hall-based sensor (AS5600) is mounted on top and can measure the rotation angle. These components are illustrated in Fig. 2c.

A fan is integrated into the experimental characterization setup to simulate flying mode with the airflow. Applying an airflow enhances the cooling process but also affects the heating energy needed for activation. However, by increasing the applied heating power, an almost adiabatic heating process can be reached with negligible heat losses. Applying high currents improves efficiency and the operational frequency by reducing the losses and the heating time.

A higher current for a shorter duration is required to achieve even higher frequencies. A new electronic circuit with an accurate current source is needed to reach high actuation frequencies with target values of several 100 Hz, paving the way for a broad range of new possible SMA applications in robotics and other fields like biomedical systems. The requirements are determined based on the following equations, which utilize the results of previously conducted experiments:

1 $$\begin{aligned} {\begin{matrix} E = R \cdot I^{2} \cdot t \\ \frac{E}{l} = \frac{\rho }{A} \cdot I ^{2} \cdot t \\ \frac{E_1}{l_1} = \frac{E_2}{l_2} \\ I^{2}_{1} \cdot t_{1} = I^{2}_{2} \cdot t_{2} \\ \end{matrix}} \end{aligned}$$

The electrical energy *E* across a resistor *R* is given by multiplying its value with the squared value of the current *I* flowing through it and the duration *t* of the current flow. The normalization of the energy equation by the length of the SMA wires *l* allows the length-independent comparison of different SMA wires. The specific resistivity $\rho$ and area *A* remain unchanged in the conducted experiments. Indices 1 and 2 refer to different experiments.

This applied approach is valid under the assumption of an adiabatic heating process with no energy losses during activation, based on Eq. (2).

2 $$\begin{aligned} E_{min}= c_{SMA} \cdot m \cdot (T_{Af} - T_{amb} ) + H \cdot m+ h_{SMA} \cdot A_{SMA} \cdot \int _{0}^{t_{on}}(T_{SMA} (t) -T_{amb}) \,dt \end{aligned}$$

The minimal required electrical energy input $E_{min}$ to reach the austenite finish temperature $T_{Af}$ is given in Eq. (2) as the contribution of three terms. The first term considers the amount of energy needed to heat the SMA element from the initial ambient temperature $T_{amb}$ to $T_{Af}$, it results from the product of the temperature difference ($T_{Af}$ - $T_{amb}$), the specific heat capacity $c_{SMA}$, and mass of the SMA element *m*. The second term is related to the latent heat, the energy required for the phase transformation as a product of the latent heat coefficient *H* and the mass. The last term represents the energy losses caused by the heat rejection into the ambient through convection. It is an integral term that considers the temperature change of the SMA element over time $T_{SMA}(t)$. The term is weighted by the factors $h_{SMA}$ the heat transfer coefficient and $A_{SMA}$ the surface of the SMA element. In an adiabatic activation process with no losses, the last term vanishes.

**Figure 6:**
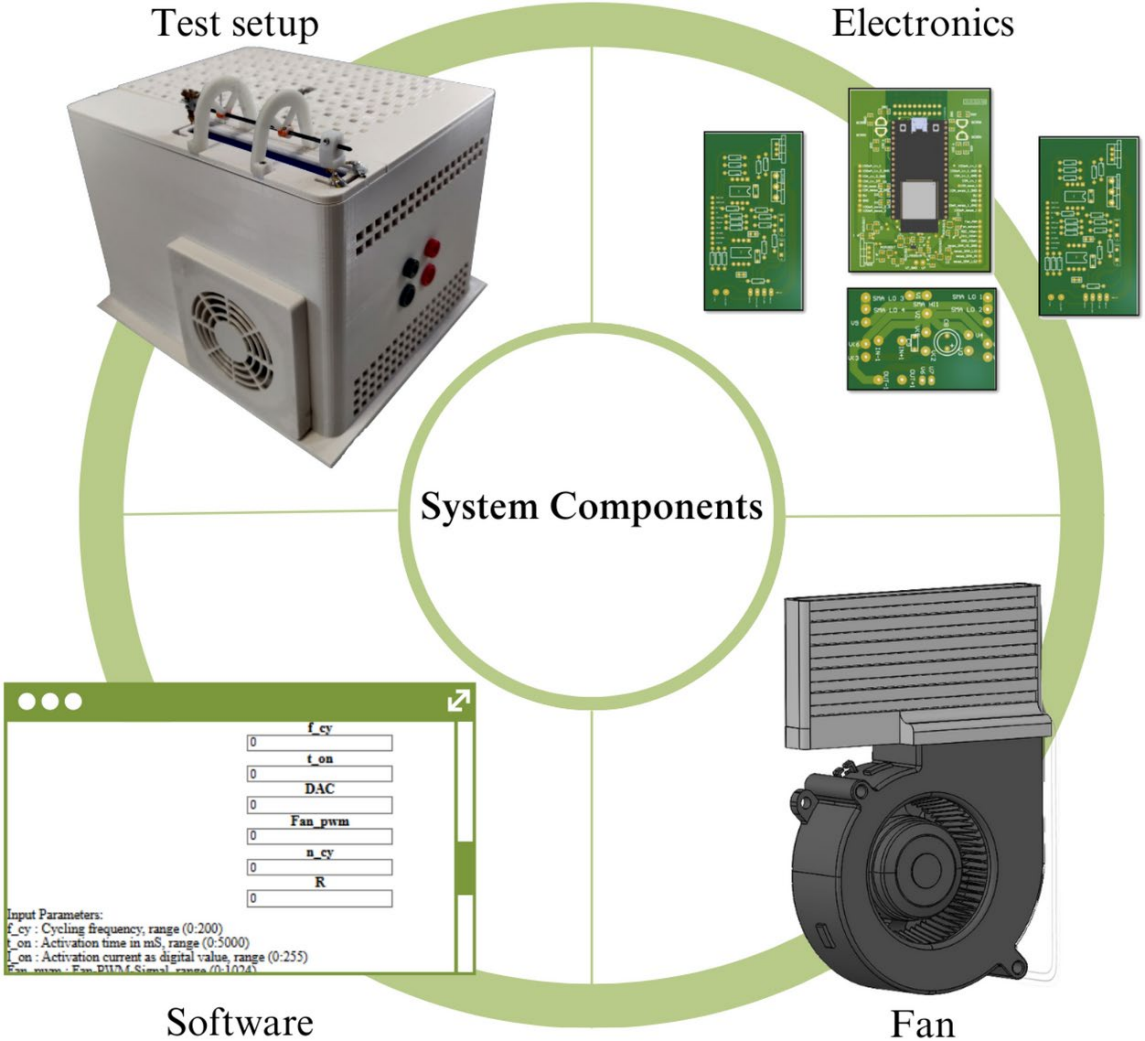
An overview of the main system components.

The developed electronic device enables continuous measurement of the resistance of the SMA bundles throughout the entire actuation time to utilize the self-sensing property of SMA. The experimental parameters can be adjusted using a WebApp (Fig. 6) and sent to the controller of the BIRAN actuators using Bluetooth. The illustrated control and measurement circuit (Fig. 6) with its microcontroller generates a control signal and measures the resistance of the SMA bundle segments throughout the entire actuation process using the four-wire method (separated voltage and current measurement). All measurement data is stored on an SD card for post-process analysis. An example of the measurement signal at 20 Hz is illustrated in Fig. 6.

### Electronic system and software

#### Current source

The current source consists of an N-Power-MOSFET (IXFH26N50P), and its gate is controlled using a precision, high slew rate operational amplifier (OPA2192). The operational amplifier compares an input voltage given by the user at its non-inverting input ($\text {U}_{\text {input}}$) with the voltage at the inverting input. The inverting input is connected to two power shunt resistors in series with a total resistance value of 0.2 $\Omega$ (PWR221T-30-R100F). The power shunt resistors are located between the source pin of the MOSFET and the ground. The circuit schematic is provided in the supplementary content. The current flowing through the load is given by:

3 $$\begin{aligned} I = \frac{U_{input}}{R_{shunt}} \end{aligned}$$

The resulting current is independent of the load, which is placed in line with the MOSFET between its drain pin and the power supply, as long as the power supply is high enough to deliver the desired current. The developed current circuit can derive currents up to 17 A with a rising time below 1 $\upmu$s for resistive load. Inductive loads can lead to delays in the rising time. The rising time is measured using the 10%- to 90% method. There are two current sources for activating the two bundle sections. In addition, two additional current sources continuously provide small measuring currents for resistance measurement of the SMA bundles.

#### Control and measuring circuit

**Figure 7:**
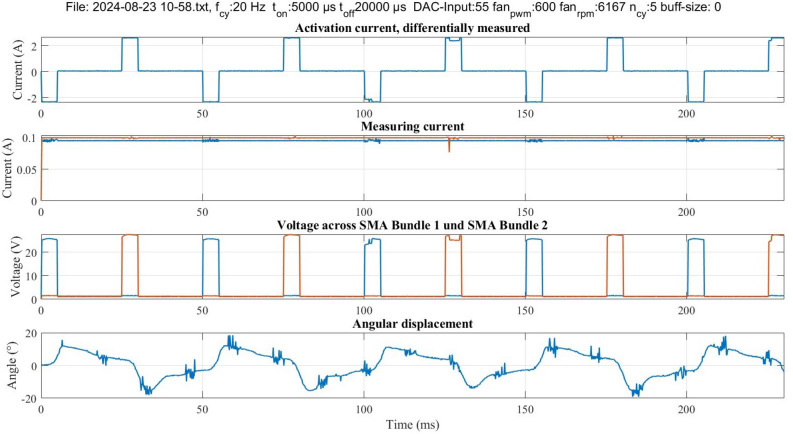
Example of the output data of an experiment. In each experiment, the control current is measured differentially, the measurement current is flowing continuously through both SMA bundles, and the third plot shows the voltage measurement across the bundles. The last plot shows the measured angle in degree.

The two control current sources are managed using a dual-core-microcontroller (ESP23). An input voltage value is defined using the Digital to Analog Converter (DAC) of the microcontroller and then switched between the voltage-controlled current sources. The microcontroller also measures the resistance of the SMA bundles to utilize the self-sensing property of SMA. To obtain accurate measurements, high-accuracy 16-bit analog-to-digital converters (ADCs) (MCP3313D-10) are used. Using 5 differential ADCs the change in the resistance of the SMA bundles can be measured during the entire actuation process, even in the non-activated state. Two differential ADCs measure the voltage difference across the bundle segments. Two differential ADCs indirectly measure the measurement currents through both bundles. The last differential ADC indirectly measures the control current through both bundles. This works using one ADC as the control current flows only through one of the bundles at the same time. The electronic system allows a sampling rate of 2 ksps. An example of measurement data is shown in Fig. 7.

#### Software

The microcontroller ESP32 possesses two cores that can operate in parallel. The time crucial tasks are implemented in core 2, which include generating the control signal and the resistance and angle measurement. For almost simultaneous measurement readings, the chip-select pins of all ADCs are pulled up by the microcontroller in the first step. By doing so, they take a sample, convert it, and save it. After pulling down the chip select pins, the ADC sends the converted value using its SPI interface to the microcontroller. The microcontroller checks before pulling down the chip select pin of the next ADC if it is time to switch between the two current sources. The microcontroller sends the saved measurement data in chunks into the SD card for post-process evaluation.

## Limitations and future work

The BIRAN actuator performs rotational actuation at high frequencies up to 200 Hz. However, a degradation in the reached rotation angle is measured towards higher frequencies. The reason for the degradation is mainly the not sufficient current applied for activation due to the limitation of the used power supply. In Fig. 4 it is shown that the SMA bundle has a resistance of $12~\Omega$. As the power supply is limited to 80 V, a maximum current of around 6.7 A can flow through the activated bundle according to Ohm’s law. Within the defined activation time, the reached heat is not sufficient to finish the phase transformation from martensite to austenite, thus the SMA bundle doesn’t fully contract. In future work, maximizing the reached angle at 200 Hz is aimed. The needed current for this is 9.8 *A* according to the minimum required activation heat calculated in previous work^25^. Besides increasing the applied electrical power, the rotation angle can be optimized by a mechanical approach based on mechanical design modification of the BIRAN actuator to shift its resonance frequency towards 200 Hz, similarly to^37^. A further approach suggests using the recently available SMA wires with a diameter smaller than 25 $\upmu$m to improve the passive convection-based cooling process by increasing the surface-to-volume ratio. Besides maximizing the rotation angle, we are also interested in examining the ability to develop compact electronics with a powerful portable power source as it aligns with the vision of developing high frequency flying SMA robots.

## Conclusion

An electrically activated bidirectional rotational antagonistic SMA actuator (BIRAN) is improved to reach higher frequencies up to 200 *Hz*, a new record in the actuation frequency of non-MEMS SMA-based actuators with meaningful displacement. At 200 *Hz* a rotation angle of $14^\circ$ is reached repeatedly. For this result, a high current, 6.7 A, is applied to shorten the heating time required for activation. At even higher currents, larger displacements are expected. A bundle of $25~\upmu m$ thick SMA wires is used in the BIRAN actuator to simultaneously support the cooling process passively by increasing the surface-to-volume ratio and maintain a sufficient force. Applying higher currents decreases the time required to reach the activation energy and saves thereby more time for cooling. If the applied currents are sufficiently high, an almost adiabatic heating process can be reached with negligible convection losses despite the high surface-to-volume ratio. To prevent overheating and damaging the SMA wires, a control strategy is implemented. In addition, a continuous airflow actively supports the cooling process and imitates the aerodynamic conditions of a flapping robot. The BIRAN actuator is bio-inspired and aims to imitate the flapping motion at higher frequencies. In future work, various approaches to reach higher rotation angles will be experimentally examined. Furthermore, the electronic system is to be downsized to realize compact SMA flying robots.

## Supplementary Information

Supplementary Figure 1.

Supplementary Figure 2.

Supplementary Figure 3.

Supplementary Video 1.

Supplementary Video 2.

Supplementary Video 3.

## Data Availability

The datasets used and/or analysed during the current study available from the corresponding author on reasonable request.
