# Supplementary figures and images for: Bidirectional rotational antagonistic shape memory alloy actuators for high-frequency artificial muscles
Source: Sci Rep. 2025 Mar 17;15:9108. doi: 10.1038/s41598-025-93209-9 (PMC11914154; doi:10.1038/s41598-025-93209-9)

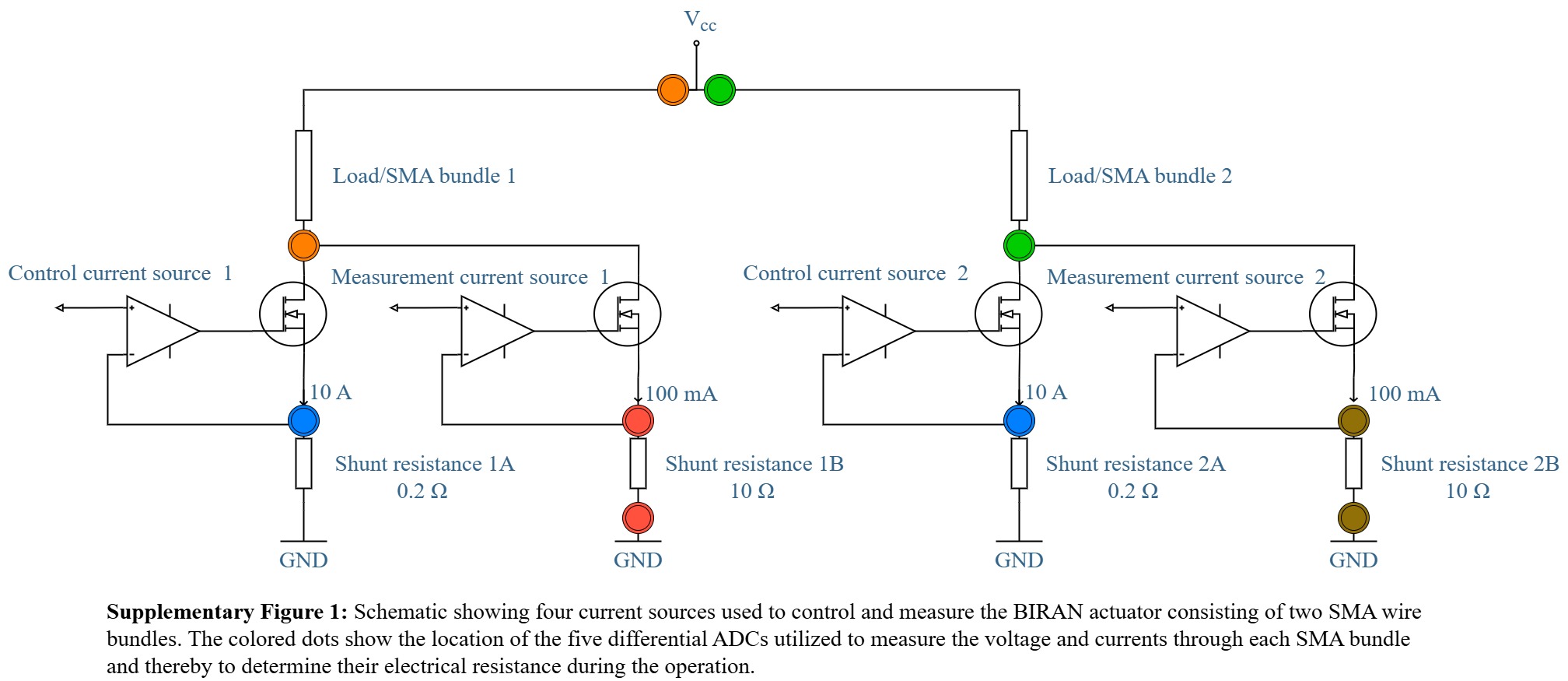

Supplement: Supplementary file 1 — Supplementary Figure 1. [file 41598_2025_93209_MOESM1_ESM.jpg]

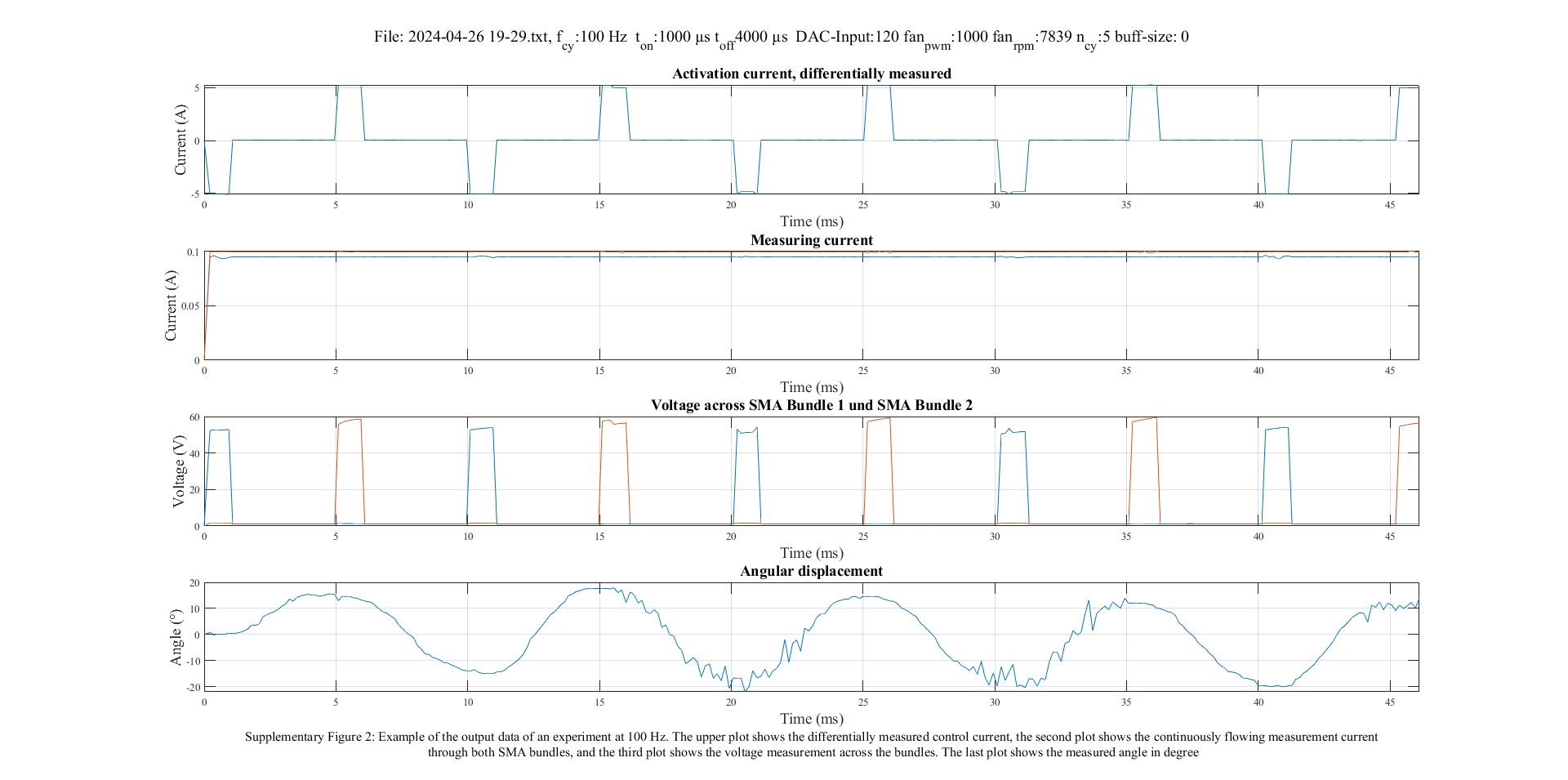

Supplement: Supplementary file 2 — Supplementary Figure 2. [file 41598_2025_93209_MOESM2_ESM.jpg]

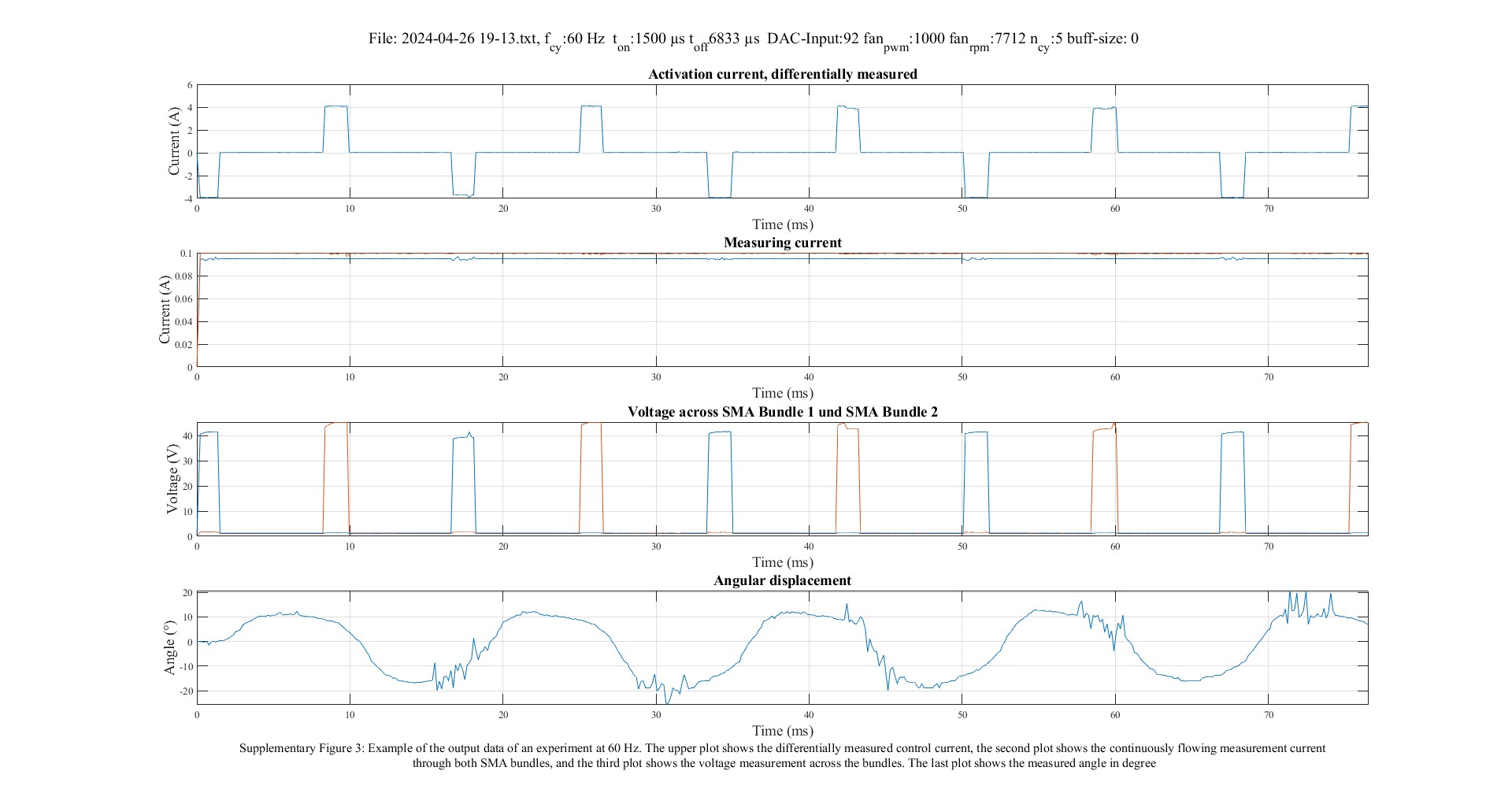

Supplement: Supplementary file 3 — Supplementary Figure 3. [file 41598_2025_93209_MOESM3_ESM.jpg]
